# Molecular Modelling of NONO and SFPQ Dimerization Process and RNA Recognition Mechanism

**DOI:** 10.3390/ijms23147626

**Published:** 2022-07-10

**Authors:** Tommaso Laurenzi, Luca Palazzolo, Elisa Taiana, Simona Saporiti, Omar Ben Mariem, Uliano Guerrini, Antonino Neri, Ivano Eberini

**Affiliations:** 1 Department of Oncology and Hemato-Oncology, University of Milan, 20122 Milan, Italy; tommaso.laurenzi@unimi.it (T.L.); elisa.taiana@unimi.it (E.T.); antonino.neri@unimi.it (A.N.); 2 Dipartimento di Scienze Farmacologiche e Biomolecolari, Università degli Studi di Milano, 20133 Milan, Italy; luca.palazzolo@unimi.it (L.P.); simona.saporiti@unimi.it (S.S.); omar.benmariem@unimi.it (O.B.M.); uliano.guerrini@unimi.it (U.G.); 3 Hematology, Fondazione Cà Granda IRCCS Policlinico, 20122 Milan, Italy; 4 Data Science Research Center (DSRC), Università degli Studi di Milano, 20122 Milan, Italy

**Keywords:** DBHS, NONO, SFPQ, structural bioinformatics

## Abstract

NONO and SFPQ are involved in multiple nuclear processes (e.g., pre-mRNA splicing, DNA repair, and transcriptional regulation). These proteins, along with NEAT1, enable paraspeckle formation, thus promoting multiple myeloma cell survival. In this paper, we investigate NONO and SFPQ dimer stability, highlighting the hetero- and homodimer structural differences, and model their interactions with RNA, simulating their binding to a polyG probe mimicking NEAT1guanine-rich regions. We demonstrated in silico that NONO::SFPQ heterodimerization is a more favorable process than homodimer formation. We also show that NONO and SFPQ RRM2 subunits are primarily required for protein–protein interactions with the other DBHS protomer. Simulation of RNA binding to NONO and SFPQ, beside validating RRM1 RNP signature importance, highlighted the role of β2 and β4 strand residues for RNA specific recognition. Moreover, we demonstrated the role of the NOPS region and other protomer’s RRM2 β2/β3 loop in strengthening the interaction with RNA. Our results, having deepened RNA and DBHS dimer interactions, could contribute to the design of small molecules to modulate the activity of these proteins. RNA-mimetics, able to selectively bind to NONO and/or SFPQ RNA-recognition site, could impair paraspeckle formation, thus representing a first step towards the discovery of drugs for multiple myeloma treatment.

## 1. Introduction

Non-POU domain-containing octamer-binding protein (NONO) and splicing factor proline- and glutamine-rich (SFPQ) are RNA-binding proteins (RBPs) belonging to the Drosophila behavior human splicing (DBHS) family. Together with paraspeckle protein 1 (PSPC-1), they are core protein components of the paraspeckles, with subnuclear bodies observed in the interchromatin space of mammalian cells placed between larger nuclear speckles and chromatin [1,2]. Although paraspeckles contain at least 60 different proteins, their biogenesis and structural integrity mainly depend on the interaction of NONO, SFPQ, and PSPC-1 homo/heterodimers with the long non-coding RNA nuclear-enriched autosomal non-coding transcripts (NEAT1) [2,3,4]. NONO, SFPQ, and PSPC-1 are involved in several nuclear processes, such as pre-mRNA splicing, DNA repair, and transcriptional regulation [5]. Indeed, the NONO::SFPQ heterodimer interacts with RNA polymerase II to promote the initiation and elongation of transcription [6,7]. Moreover, the NONO::SFPQ heterodimer is needed early for spliceosome formation [8,9,10] and has a role in DNA double-strand break repair through non-homologous end-joining (NHEJ) [11]. The deregulation of SFPQ and NONO is reported to induce senescence [12].

In vitro studies demonstrated that DBHS protein interaction mediates specific functions, depending on the cellular context or developmental stage, by modulating protein expression [13]. Indeed, the mechanism of paraspeckle formation has been demonstrated to be important for multiple myeloma cell survival and proliferation [14]. Thus, studying the DBHS protein dimerization and complex formation with NEAT1 could be relevant for the treatment of this incurable disease. DBHS protein expression is regulated during prostate cancer progression and spermatogenesis [15,16]. Moreover, other research groups have pointed out their involvement in neurodegenerative diseases [17]. NONO, SFPQ, and PSPC-1 share more than 70% sequence identity within the common core region of approximately 300 amino acids (named the DBHS domain). This region comprises two RNA recognition motif domains (RRM1 and RRM2), a conserved region named the NONO/paraspeckle (NOPS) domain and coiled-coil region. The coiled-coil expands beyond the DBHS region to create the ɑ-helices necessary for protein polymerization [5]. In addition, these proteins contain a C-terminal nuclear localization signal and specific low complexity regions: proline/glutamine- and glycine-rich regions in SFPQ, histidine/glutamine-rich and proline-rich regions in NONO, and proline/alanine- and glycine-rich regions in PSPC1 [5]. The crystal structures of the DBHS proteins provide molecular details of the homo- and hetero-dimerization process [5,13,18,19,20]. The homo- and hetero-dimerization is mediated by RRM2, NOPS, and part of the coiled-coil regions, which are also required for structural stability [5]. The consensus sequence for oligomerization is comprised between residues 497–596 (SFPQ) or 268–372 (NONO), resulting in an antiparallel coiled-coil interaction [5]. In SFPQ, the coiled-coil domain mediates the interaction with NONO, producing a tetramer of two SFPQ and two NONO subunits [5,21].

Until today, few data are available regarding the predisposition of DBHS proteins to homo- and hetero-dimerization. In vivo studies suggest that the NONO::SFPQ complex would be preferred among all the DBHS complexes [22]. The preferential heterodimerization is likely to depend on the structures observed at the dimer interfaces encompassing all four domains [20]. Accordingly, the aim of the present paper is to deepen the molecular bases of the NONO::SFPQ heterodimer interaction and evaluate their predisposition to form hetero- or homo-dimers, based on their interaction energy.

## 2. Results and Discussion

### 2.1. Comparison of NONO and SFPQ Dimer Stability

To equilibrate the crystallographic structures and analyze the dynamical behaviors of the NONO::NONO and SFPQ::SFPQ homodimers and NONO:SFPQ heterodimer, we carried out three 3 μs MD simulations. The evaluation of the dimer stability during the simulations was based on both geometric and energetic parameters, as described below.

**Figure 1:**
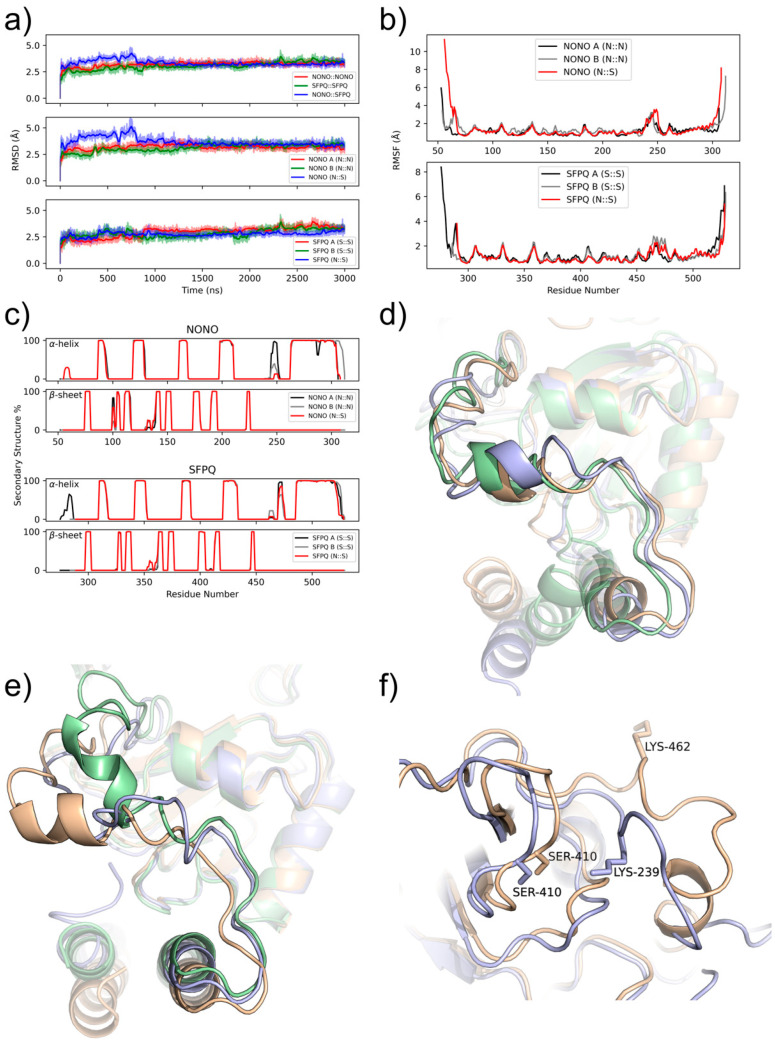
NONO and SFPQ dimers are highly stable. Through MD, we assessed NONO and SFPQ dimers stability. (**a**) RMSD converges to a stable plateau of 2.5 Å within 1 µs for all systems. RMSD is reported for dimers (top) and individual chains of NONO (middle) and SFPQ (bottom). (**b**) RMSF is reported for individual chains in all dimer combinations; mobile regions overlap in all systems, with higher fluctuations at the N- and C-termini and NOPS region. (**c**) Secondary structure % over simulation time: ɑ helix (top), β strands (bottom). (**d**,**e**) NOPS helix in NONO and SFPQ superposed structures: homodimer (yellow and green), heterodimer (blue). (**f**) SFPQ homo- (yellow) and heterodimer (blue) interaction between RRM2 β2/β3 loop and NOPS.

The general stability of the three dimers is confirmed by the tendency of the MD simulations to reach convergence within a short simulated time frame. In detail, the RMSD values reach a plateau around 2.5 Å for all dimers within 1 µs (Figure 1a). However, the NONO::SFPQ heterodimer had an higher RMSD for the very first part of the simulation. To understand the bases of this conformational rearrangement, we analyzed the stability of the single monomers, revealing that the NONO in the heterodimer crystallographic structure required a longer time to reach conformational equilibrium (Figure 1a). This is probably due to the different interactions between NONO and SFPQ, with respect to the NONO homodimer. However, this behavior may also depend on the experimental conditions, which are not yet publicly available at the time of writing, in which the heterodimer crystals were produced.

To better describe the dynamical behaviors of the NONO and SFPQ proteins within the homo- and hetero-dimers, we calculated the root mean square fluctuations (RMSF) of each residue within the simulations (Figure 1b). Protein secondary structure elements (SSEs) were also monitored throughout the simulation. Figure 1c reports SSE distribution via the residue index, which summarizes the SSE composition for each trajectory frame over the course of the simulation time. Globally, the secondary structure of the dimers is conserved during the MD simulations.

When taken together, these data show that, for all dimers, the lowest RMSF values are associated with α-helices and β-sheets, while the highest values are associated both with random coils and N- and C-termini.

From these analyses, we detected a highly mobile stretch of residues between RRM2 and coiled-coil regions (NOPS). These residues (245–250, NONO; 468–473, SFPQ) form an α-helix facing the RRM2 of the other monomer (Figure 1d,e). Note that this region is missing from the crystallographic structure of the NONO::SFPQ heterodimer, which was modelled during the protein preparation procedure. However, despite the long MD simulation time, this region did not fold as an α-helix. Moreover, we observed highly mobile residues in the SFPQ homodimer within the β2/β3 loop (residues 405–413) of the RRM2. These residues are more stable in the heterodimer because of an interaction between Ser410 of SFPQ with Lys239 of NONO that would not be possible in the homodimer, where the corresponding residue Lys462 of SFPQ is too far from Ser410 (Figure 1f).

**Figure 2:**
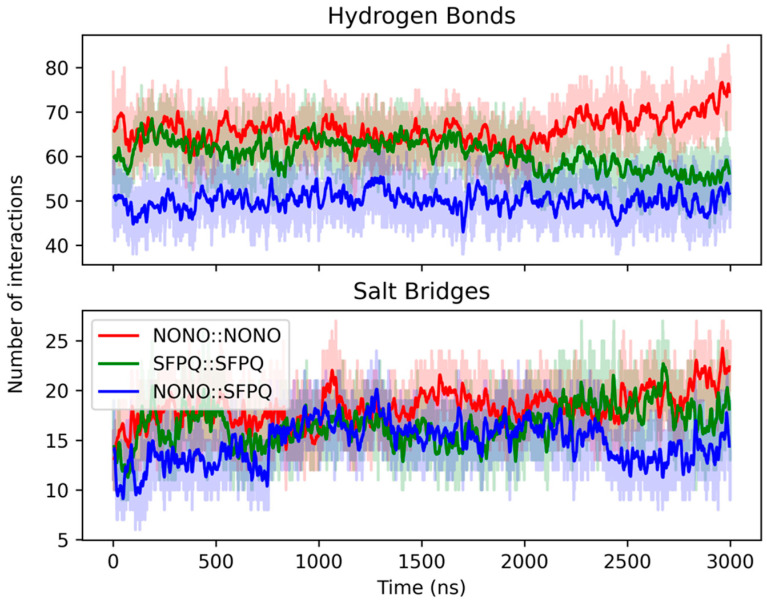
MD polar interactions. NONO::NONO (red), SFPQ::SFPQ (green), NONO::SFPQ (blue). Total number of polar interactions and their fluctuations was stable in all systems. The NONO::SFPQ heterodimer displayed a smaller count of total hydrogen bonds, compared to the other systems.

By analyzing the molecular interactions throughout the simulations, we observed that all the dimers conserve over 50 H-bond interactions during the MD simulations (Figure 2). In particular, the NONO::NONO dimer has a mean of 66 ± 4 H-bonds, while SFPQ::SFPQ and NONO::SFPQ dimers have a mean of 60 ± 4 and 50 ± 4 H-bonds, respectively. A similar distribution can be observed for salt bridges, ranging between 18 ± 2 for NONO::NONO, 17 ± 3 for SFPQ::SFPQ and 15 ± 3 for NONO::SFPQ. Overall, the homodimers displayed an higher number of polar interactions, compared to the heterodimer, with NONO::NONO having the highest number.

However, the number of polar interactions is not sufficient to fully characterize the interaction profiles between the homo- and hetero-dimers, as solvation effect and hydrophobic and short-range interactions may significantly affect dimer formation. For this reason, we calculated the dimer formation energy, in order to evaluate whether the homo-/hetero-dimerization of NONO and SFPQ is a thermodynamically favored process, as well as whether there could be any inconsistency, in terms of energy, between complexes. Dimer formation energy was calculated by comparing the energetic features between MD simulations of DBHS monomers and dimers, resulting in the energy difference between the reactants and products of the dimerization process. Being performed on explicit solvent MD simulations, this procedure should implicitly capture the role of solvation in dimers stability. More details on dimer formation energy calculation are described in Section 3. Materials and Methods.

**Figure 3:**
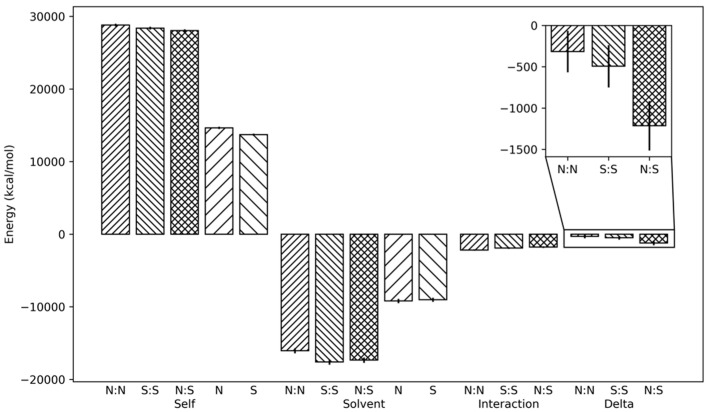
The NONO::SFPQ heterodimer formation is thermodynamically favored, when compared to NONO and SFPQ homodimers. Decomposition of interaction energies: protein internal energy, protein::solvent interaction, protein::protein interaction. Zoomed pane is dimer formation energy. Energy values are averaged over the stable last 50% of MD trajectories, error bars represent the standard deviation.

Figure 3 reports the average dimer formation energies: both NONO and SFPQ homodimers have statistically comparable dimer formation energies, despite having a higher number of polar interactions between monomers, with respect to the NONO::SFPQ heterodimer, and the latter shows the most negative energy value (approx. −1200 kcal/mol). This result may contribute to explaining the experimental results at an atomistic level by showing a higher tendency of DBS proteins to heterodimerize, compared to homodimerization [20,22]. Globally, this analysis shows that the formation of all the simulated complexes is favored from a thermodynamic point of view, which also confirms the previous observations of others [20,22].

### 2.2. NONO and SFPQ Dimers Have Different Interaction Patterns

**Figure 4:**
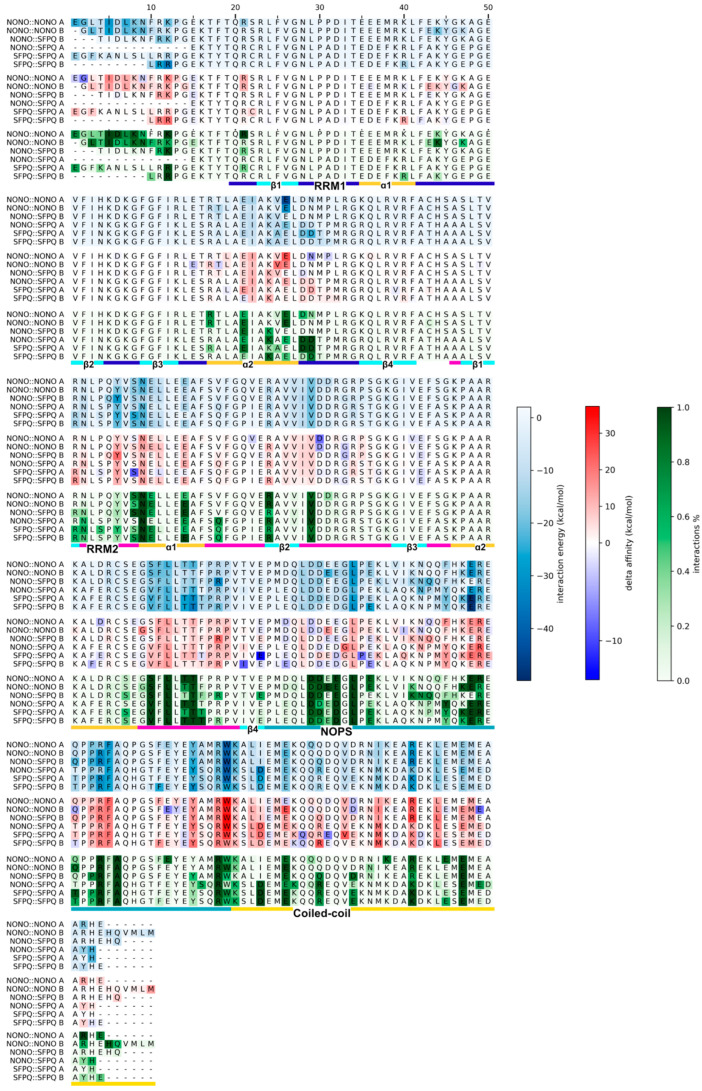
Comparison of MD interactions, single-point interaction energies, and alanine scan delta affinity. Time-persistence of hydrogen bonds, salt bridges, and pi–pi and pi–cation interactions was measured along MD trajectories, as a fraction of the simulation time. Single-point energy contributions and alanine scan delta affinity were computed on the MD medoids. The three complementary methodologies highlight the key residues responsible for inter-chain protein::protein interactions.

To investigate the residues involved in the dimerization process, we integrated molecular dynamics simulation interaction analyses with single point potential energy calculations and an in silico alanine scan. We recorded how long each residue of each monomer was involved in interactions with its binding partner, which was averaged over the simulated time, considering the hydrogen bonds, salt bridges, and Pi–Pi and Pi–cation interactions. Cluster analysis of the trajectories allowed us to identify the most stable conformations by extracting the medoid of the largest clusters. On these configurations, we performed a single point energy calculation and decomposed the potential interaction energies at the residue level. On the same structures, we then performed an in silico alanine scan experiment, in order to individuate the hot spot residues necessary for the dimer’s stability. Figure 4 combines the results of the abovementioned analyses, remarking on a high level of consistency between the results of these different approaches.

**Figure 5:**
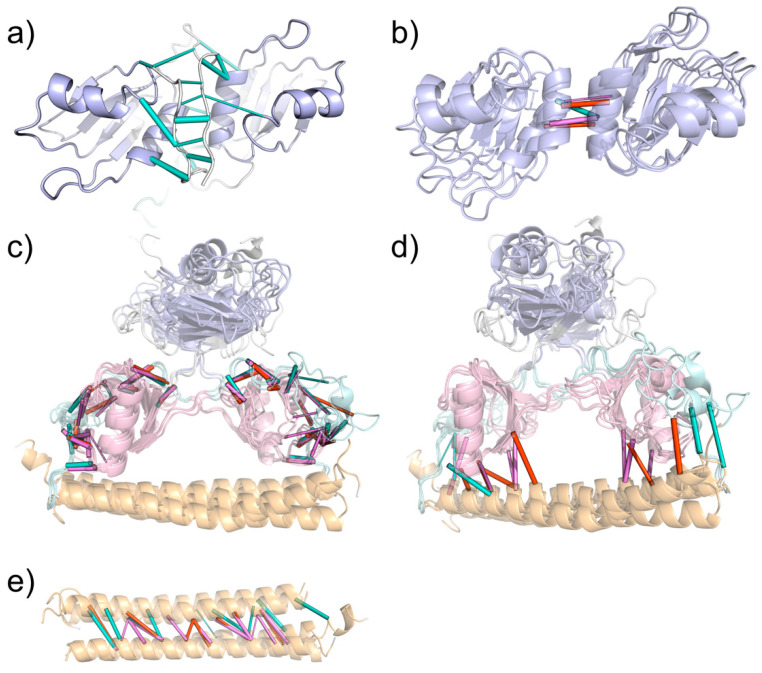
Molecular dynamics interactions network. (**a**) Beta-clasp interactions between N-termini in NONO::NONO. (**b**) Backbone-to-backbone interactions between RRM1s helix 2. (**c**) RRM2-NOPS interactions. (**d**) RRM2 and NOPS interactions with coiled-coil. (**e**) Coil-coil interactions. Cartoon colors: RRM1 (blue), RRM2 (pink), NOPS (light blue), coiled-coil (yellow). Interaction tube colors: NONO::SFPQ (red), NONO::NONO (cyan), SFPQ::SFPQ (magenta).

First, we identified persistent backbone-to-backbone hydrogen bond interactions within the N-terminal loops for only the NONO::NONO homodimer (Figure 5a), in accordance with the antiparallel β-clasp interactions, as described by Knott et al. [23].

In all the investigated dimers, we were able to observe RRM1 domains interacting with each other at the level of ɑ-helix 2 (Figure 5b), both through polar and hydrophobic interactions. It is interesting to note that the contribution of hydrophobic interactions could not be established by MD alone but needed the integration with the single-point and alanine scan analyses. Only SFPQ-containing dimers can form the interaction between Asp354 and Lys64 (NONO) or Arg287 (SFPQ), which map to the same position in the sequence alignment (column 12).

Overall, multiple (conserved) interactions could be found between the RRM2s of one monomer and the coiled-coil and NOPS domains of the other (Figure 5c,d). Specifically, we were able to observe the conserved interactions between the RRM2 β1/ɑ1 loop and coiled-coil domains of the other monomers (Figure 5). Additionally, RRM2 ɑ1 strongly interacts with the NOPS small ɑ-helix. Moreover, MD analysis captured the specific interactions of the SFPQ dimers between the SFPQ Glu393 and NONO Pro255/SFPQ Pro478 backbone. Conserved interactions are formed between the RRM2 β2/β3 and NOPS loops: Val (SFPQ 404, NONO 181)—Asp (SFPQ 454, NONO 231) and Arg (SFPQ 399, NONO 176)—Asp (SFPQ 455, NONO 232). The loop between RRM2 ɑ2 and β4 strongly interacts with the NOPS region, and Ser212 (NONO) and Val435 (SFPQ) form backbone–backbone hydrogen bonds with conserved Ala258 (NONO) or Ala481 (SFPQ). In the same region, backbone interactions are also present between conserved Leu214 (NONO) or Leu437 (SFPQ) and Arg256 (NONO) or Arg479 (SFPQ). Moreover, the backbone of Arg256 (NONO) and Arg479 (SFPQ) can form other multiple interactions with RRM2 at the level of the ɑ2/β4 loop, with two adjacent threonine residues: Thr216-217 (NONO) and Thr439-440 (SFPQ).

As expected, coiled-coil helices primarily interact with themselves (Figure 5e); however interactions may also occur between coiled-coil residues and RRM2s, preferentially in the SFPQ-containing dimers via Asp498 and Lys495, whilst, in NONO, only Lys272 can interact with the other protomer RRM2 for more than 30% of the simulated time. Of all systems, the NONO::SFPQ heterodimer has the largest number of coiled-coil::RRM2 interactions, which formed between SFPQ coiled-coil and NONO RRM2. Additionally, in SFPQ-containing dimers, we observed the highest number of coil-to-coil interactions within the coiled-coil domains.

The analysis of hydrophobic interactions (Supplementary Figure S1) also revealed that the NONO::SFPQ and SFPQ::SFPQ dimers can form hydrophobic interactions between the facing RRM1 ɑ2 helices; however, in the NONO::NONO homodimer, these helices form hydrophobic interactions with the longer N-termini, which are responsible for the β-clasp structure described above. In addition to this, all dimers show common hydrophobic interaction patterns, which, similarly to the previously described inter-chain polar interactions, are mainly present between the antiparallel coiled-coil domains, RRM2 and NOPS, and RRM2 and coiled-coil, thus availing the central role of RRM2s in stabilizing the dimerization.

### 2.3. Analysis of NONO and SFPQ Dimer Interactions with RNA

**Figure 6:**
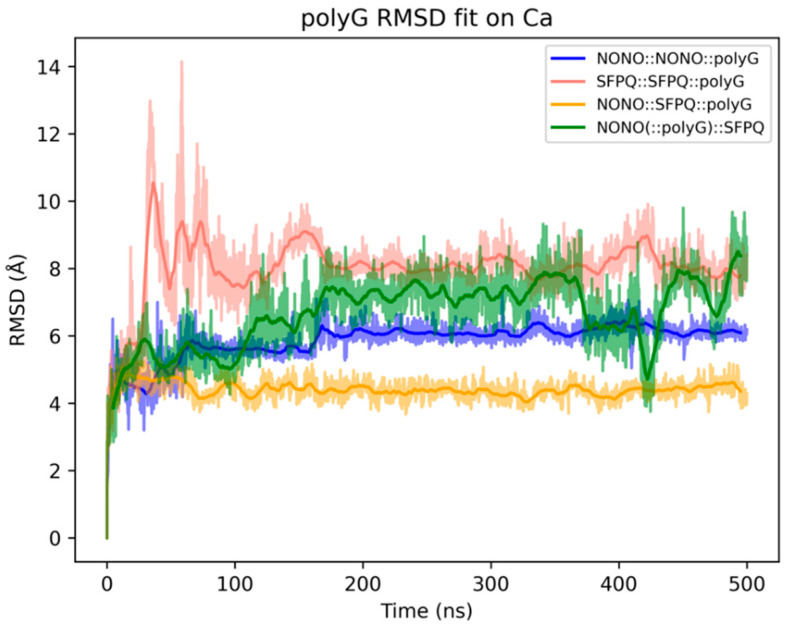
Stability of DBHS::polyG dimers during MD simulation. RMSD was calculated on polyG atoms after fitting each frame to the solute ɑ-carbons.

We investigated the recognition mechanism between RRM1s and RNA by simulating the dynamics of homo- and hetero-dimers in complex with a short guanosine pentanucleotide mimicking the NEAT1 guanine-rich regions [23,24]. We submitted four DBHS::polyG complexes to 500 ns MD simulation: NONO::NONO(::polyG), SFPQ::SFPQ(::polyG), NONO(::polyG)::SFPQ, and NONO::SFPQ(::polyG). Complexes were built, as described in the materials and methods, using the binding mode of the RNA co-crystalized with CUGBP1 (PDB: 3NNA) as a reference [25]. We first analyzed the evolution of the initial binding mode towards more stable configurations by monitoring polyG RMSD after fitting on the protein ɑ carbons (Figure 6). NONO::SFPQ(::polyG) and NONO::NONO(::polyG) show a remarkably stable binding mode, conserved throughout the entire simulation; NONO(::polyG)::SFPQ and SFPQ::SFPQ(::polyG) have an oscillating polyG RMSD profile. It’s noteworthy, however, that, despite some fluctuations, all the largest trajectory clusters extracted with the same RMSD metric display a similar RRM1::polyG binding mode (Figure 6), and the protein::RNA interaction energy profiles remain constant throughout the simulations (Supplementary Figure S2), thus indicating persisting favorable interactions.

**Figure 7:**
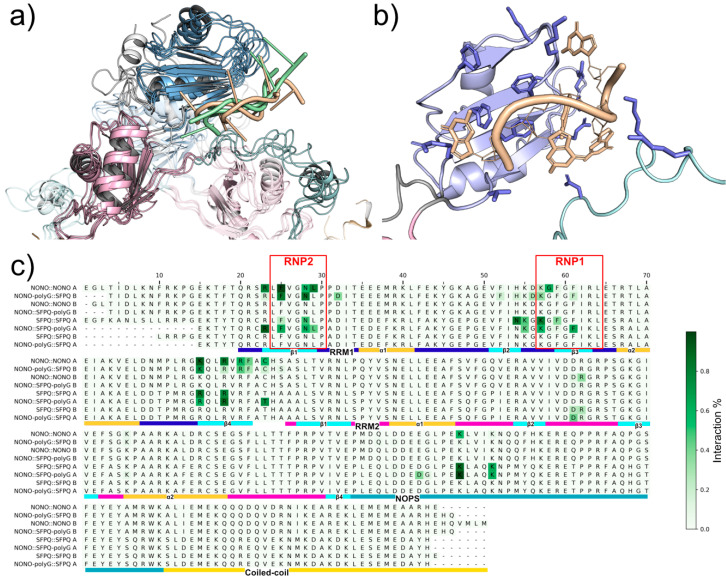
DBHS::polyG MD interactions. (**a**) Comparison of DBHS::polyG binding modes: NONO-bound (green), SFPQ-bound (yellow). (**b**) Representative binding mode of SFPQ bound to polyG in NONO::SFPQ, which has the most stable configuration; interacting residues are represented with blue sticks; polyG (yellow). (**c**) Interaction existence over simulation time: most DBHS::polyG interactions happen within RRM1s; however, the other chain β2/β3 loop can assist with RNA binding. Sequence ordering: NONO bound to polyG in N::N (1) and N::S (2); NONO unbound in N::N (3) and N::S (4); SFPQ bound to polyG in S::S (5) and N::S (6); SFPQ unbound in S::S (7) and N::S (8).

By recording per-residue protein::RNA interactions along trajectories, we were able to identify the conserved residues that are likely involved in RNA recognition (Figure 7, Supplementary Table S1). Both NONO and SFPQ RRM1s contain two conserved RNP motifs, as described in [26]. Within these motifs, RNP1 and RNP2, Phe residues are known to play a pivotal role in RNA recognition by intercalating with nucleobases. As expected, we were able to confirm such interactions with our MD simulations, where NONO Phe111, Phe113 (RNP1), and Phe77 (RNP2) and SFPQ Phe334, Phe336 (RNP1), and Phe300 (RNP2) form stable Pi–Pi interactions with polyG guanosines. Notably, we were able to observe NONO::RNA interactions with Phe104, a “fourth” Phe located within the RRM1 β2 strand. This residue may be required for specific recognition, and it may increase RNA binding affinity in NONO-containing dimers. Polar amino acids within the RRM1 region can further contribute to RNA recognition by forming hydrogen bonds with nucleobases, while positively charged residues, such as arginine and lysine, can stabilize RNA phosphate backbone via salt-bridges. These interactions were observed within the β1 strand (RNP2) and β2/β3 loop (RNP1), as well as within the β4 strand. We also identified interactions between RNA backbone and lysins in the NOPS region that were not previously hypothesized. In fact, experimental evidence supports the functional requirement of the NOPS domain for RNA binding [27]; however, this was speculated to be an indirect consequence of NOPS being necessary for dimerization [19]. Here, we show that the NOPS domain may be required directly for RNA binding. While we speculate that RRM2s do not serve for primary RNA interaction, we show that the RRM2s β2/β3 loop of the other protomer (the one not interacting directly via RRM1s) may assist in RNA binding by forming salt bridges between RNA backbone phosphate and Arg184 (NONO) or Arg407 (SFPQ).

## 3. Materials and Methods

### 3.1. Structure Preparation

The three-dimensional crystallographic structures of the NONO::NONO (PDB ID: 5IFM-1) and SFPQ::SFPQ (PDB ID: 6OWJ) homodimers and NONO::SFPQ (PDB ID: 7LRQ) heterodimer (PDB, www.rcsb.org) were subjected to a pre-processing procedure consisting of the following steps: (i) addition of ACE and NMA capping groups to N- and C- termini, (ii) addition of hydrogens and optimization of the protonation state using PROPKA [28], (iii) prediction of missing side chains positions and filling of short loops using Prime (Prime, Schrödinger, LLC, New York, NY, 2021), followed by (iv) final refinement with a restrained minimization on heavy atoms.

### 3.2. Molecular Dynamics (MD)

Molecular dynamics (MD) simulations were carried out with Desmond (Schrödinger 2019-04, D. E. Shaw Research, New York, NY; Schrödinger, New York, NY) [29]. Systems were built using the Maestro graphical interface and solvated with SPC (simple point-charge) water molecules in a cubic box with faces at a distance of 10 Å from the solute. Sodium or chlorine ions were used to reach electroneutrality; sodium chloride was also added to a 0.15 M concentration. Systems were then parameterized using Schrodinger OPLS3e forcefield and equilibrated with a standard protocol consisting of low-temperature NVT Brownian dynamics with restraints on solute heavy atoms, a heating-up stage in NPT Langevin, and a final pre-production stage in NVT Langevin, with progressive removal of restraints. Production MDs were run in isothermal–isobaric ensemble at standard temperature of 300 K using Nose–Hoover thermostat and Martyna–Tobias–Klein piston, with simulation times of 3 µs for dimers, 1 µs for monomers, and 500 ns for polyG-bound dimers.

Trajectory energetic and geometric data were extracted using the Desmond *vrun* module and Schrodinger python API. An in-house Python implementation of the GROMOS clustering algorithm [30] was used to perform trajectory frame clustering over the MD simulations. Distances for clustering were computed based on the root mean square deviation (RMSD) matrix of the proteins’ ɑ carbons for each frame of the aligned trajectory.

### 3.3. Interactions Analysis

Protein::protein and protein::RNA interactions were calculated using the Schrödinger Python API to analyze MD trajectories. Measured interactions include hydrogen-bonds, salt bridges, and pi–pi and pi–cation interactions. Hydrophobic interactions were measured on MD medoids using a protein::protein interaction analysis web server [31]. Per-residue protein::protein interaction energies were measured on MD medoids with Prime. Protein::RNA binding free energies and per-residue contributions were also measured on MD medoids via MM/GB-SA (Prime).

### 3.4. Dimer Formation Energy Calculation

For each dimer, three trajectories were considered: the dimer itself and two constituting monomers (or one monomer counted twice, in case of homodimers). For each chain of these systems, we computed the protein–solvent (including ions) and protein–protein interaction energies (in case of dimeric systems). We also computed the internal energy of each protein chain to keep account of the energy stored or released during its rearrangement. We then defined dimer formation energy ΔE, as in the following equation:

(1) $$\Delta E=E_{A:B}^{self}+E_{A:B}^{solv}+E_{A:B}^{inter}-\left(E_{A}^{self}+E_{A}^{solv}+E_{B}^{self}+E_{B}^{solv}\right)$$

where A and B indicate the two chains of the dimer.

This represents the energy balance of the transition from two separate monomers to the dimer configuration and, when negative, indicates a favorable process. For this calculation, we considered only the second (equilibrated) half of the trajectories.

### 3.5. In Silico Alanine Scan

In silico mutagenesis was performed to produce an alanine scan of each protomer in the NONO and SFPQ homo- and hetero-dimers. The methodology was carried out with Prime (Prime, Schrödinger, New York, NY). One at a time, each residue was mutated into alanine, and all residues within 6 Å from the mutation site were minimized; finally, the interaction energy between dimer protomers was computed and compared to the *wt* complex, reporting it as an ΔAffinity value.

### 3.6. DBS::PolyG Model Construction

To provide an initial guess for the RNA-bound structure, we aligned the equilibrated structures (that is, the medoids of the most populated clusters within the equilibration 3 µs MD of each system; medoids were then subject to energy minimization with MacroModel using Polak-Ribier Conjugate Gradient with a gradient threshold of 0.05 kJ mol^−1^ Å^−1^) of NONO and SFPQ RRM1s in both homo- and heterodimers to the structure of CUG-binding protein 1 (CUGBP1) in complex with RNA (PDB: 3NNA). Then, we built the polyG pentaribonucleotide from scratch using the Maestro 3D Builder graphical interface and aligned it to the co-crystalized RNA molecule in 3NNA (which is now aligned with NONO and SFPQ RRM1s) using Maestro flexible ligand alignment tool. Finally, we merged the structures of the homo- and heterodimers with the aligned pentanucleotide. This procedure yielded four models of NONO and SFPQ homo/heterodimers, with an RNA molecule proximal to each RRM1: NONO::NONO(::polyG), SFPQ::SFPQ(::polyG), NONO(::polyG)::SFPQ, and NONO::SFPQ(::polyG). These models were then refined by energy-minimizing residues, within 6 Å from the RNA ligand, using Prime implementation of MM/GBSA, and submitted to 500 ns molecular dynamics.

## 4. Conclusions

NONO and SFPQ are involved in multiple nuclear processes, such as pre-mRNA splicing, DNA repair, and transcriptional regulation. These proteins, along with the lncRNA NEAT1, are required for paraspeckle formation, i.e., nuclear structures that were shown to promote survival in multiple myeloma cancer cells. In the present paper, we investigate the stability of NONO and SFPQ dimers, highlighting the differences between hetero- and homodimers at a structural level, and propose a model of their interactions with RNA, simulating their binding to a guanosine pentanucleotide (polyG) probe that mimics the guanine-rich regions of NEAT1.

By performing MD simulations and energy calculations on NONO::NONO, SFPQ::SFPQ, and NONO::SFPQ dimers, we were able to confirm dimer stability and the different interactions between homo and heterodimers; in particular, we show that the formation of the NONO::SFPQ heterodimer is a more favorable process, compared to homodimers formation. Although our models are truncated at the N-termini, due to the lack of full-length crystallographic structures, and other phenomena may regulate hetero/homodimerization processes in vivo, our findings are in accordance with the experimental results showing a higher abundancy of DBS proteins heterodimers, compared to homodimers.

We also hypothesize that NONO and SFPQ RRM2s are not required for primary RNA interaction. In fact, RRM2s lack the typical RNP signature; instead, we show that the RRM2 residues that are in the place of the RNP signature are mostly involved in protein–protein interactions with the other DBHS protomer; meaning that RNA binding is primarily due to RRM1s.

The simulation of RNA binding to NONO and SFPQ validated the importance of the RRM1 RNP signature but also highlighted other key residues in β2 and β4 strands that could be required for specific recognition of RNA sequences. Moreover, we show that the NOPS region and other protomer’s RRM2 β2/β3 loop may also be required to strengthen RNA interactions.

Our results shed light on the interactions between RNA and DBHS dimers, which will be required for the rational design of small molecules that are able to modulate the activity of these proteins. In fact, the follow-up of this work will be aimed at the pharmacological disruption of NEAT1 binding using RNA-mimetics, which are able to selectively bind to NONO and/or SFPQ RNA-recognition site, impairing paraspeckle formation, thus being a first step towards the rational design of new drugs for the treatment of multiple myeloma.

## Supplementary Materials

The following supporting information can be downloaded at: https://www.mdpi.com/article/10.3390/ijms23147626/s1.
