# Supplementary material for: Molecular Modelling of NONO and SFPQ Dimerization Process and RNA Recognition Mechanism
Source: Int J Mol Sci. 2022 Jul 10;23(14):7626. doi: 10.3390/ijms23147626 (PMC9324803; doi:10.3390/ijms23147626)
Supplement: Supplementary file 1 [file ijms-23-07626-s001.zip › ijms-1751162-supplementary.pdf]

Molecular modelling of NONO and SFPQ dimerization process and RNA recognition mechanism.

Tommaso Laurenzi[1], *Luca Palazzolo*[2], *Elisa Taiana*[1,3], *Simona Saporiti*[2], *Omar Ben Mariem*[2], *Uliano Guerrini*[2], *Antonino Neri*[1,†], *Ivano Eberini*[2,4,†,*]

[1] Department of Oncology and Hemato-oncology, University of Milan, Milan, Italy
[2] Dipartimento di Scienze Farmacologiche e Biomolecolari, Università degli Studi di Milano, Milan, Italy
[3] Hematology, Fondazione Cà Granda IRCCS Policlinico, Milan, Italy
[4] Data Science Research Center (DSRC), Università degli Studi di Milano, Milan, Italy
[†] These authors contributed equally.
[*] Correspondence: ivano.eberini@unimi.it; Tel.: +39 02 50318256

Supplementary Data

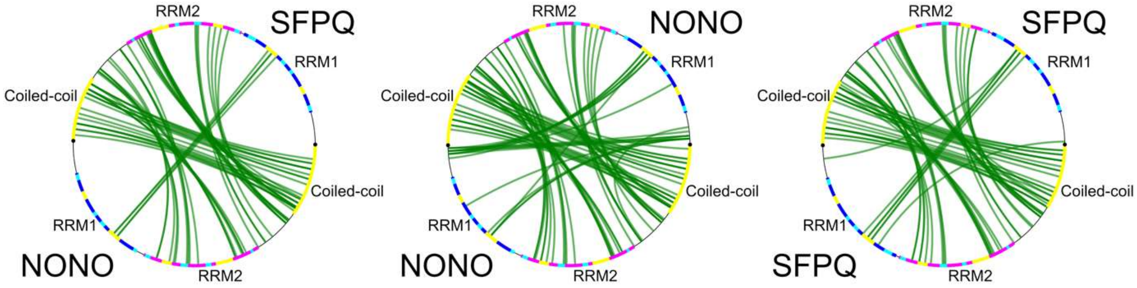

Figure 1. Hydrophobic interactions chord plot. Hydrophobic interactions are clearly visible between RRM1 alpha 2 helices and between coiled-coil domains. In NONO::NONO RRM1 alpha-2 helices interact with the longer N-termini. RRM2s interact both with NOPS and coiled-coil. Alpha helices (yellow), beta strands (cyan).

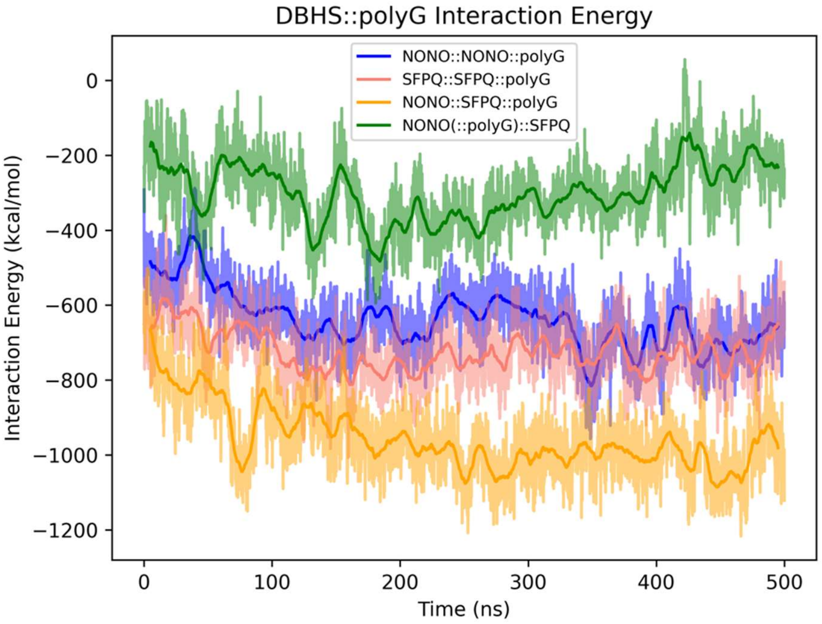

Figure 2. Interaction energy between NONO-SFPQ dimers and polyG probe calculated during MD simulations. The NONO::SFPQ::polyG

Table 1. Per-residue energy contribution to RNA binding for NONO and SFPQ computed with MM/GB-SA. Because calculations were performed on the medoids of the most populated clusters, not all interactions measured along the entire simulations are present.

**NONO**							
Bound				Unbound			
NONO::NONO(::polyG)		NONO(::polyG)::SFPQ		NONO::NONO(::polyG)		NONO::SFPQ(::polyG)	
ARG 140	-9.013	PHE 77	-5.923	ARG 184	-0.852	ARG 184	-1.669
PHE 77	-6.329	PHE 111	-5.108	LYS 64	-0.798	GLY 185	-0.595
ARG 142	-5.194	LYS 137	-4.76	ARG 186	-0.653	LYS 64	-0.552
LYS 137	-3.781	LYS 109	-4.726	ARG 176	-0.573	ARG 176	-0.524
ALA 144	-2.925	GLY 79	-4.467			ARG 184	-1.669
PHE 111	-2.863	CYS 145	-3.527			GLY 185	-0.595
LYS 109	-2.451	ARG 142	-2.658			LYS 64	-0.552
ARG 75	-2.391	PRO 82	-2.293			ARG 176	-0.524
PHE 143	-2.189	PHE 143	-2.207				
PHE 113	-2.161	PRO 83	-2.074				
GLY 79	-2.068						
**SFPQ**							
Bound				Unbound			
SFPQ::SFPQ(::polyG)		NONO::SFPQ(::polyG)		SFPQ::SFPQ(::polyG)		NONO(::polyG)::SFPQ	
LYS 332	-11.38	ARG 363	-8.081	ARG 407	-5.834	ARG 407	-2.411
PHE 334	-6.097	LYS 462	-7.692	ARG 409	-0.804	ASP 406	-1.82
LYS 462	-5.797	LYS 332	-5.597	LYS 291	-0.562	ARG 409	-0.673
ARG 363	-4.937	ARG 298	-5.278	ARG 287	-0.54	ARG 399	-0.454
ASN 329	-4.486	PHE 334	-5.174				
ARG 360	-4.275	PHE 300	-4.745				
GLY 302	-3.836	LYS 330	-4.25				
LYS 466	-3.66	PHE 336	-4.144				

ASN 303	-2.673	THR 368	-3.917				
		LYS 466	-3.847				
		GLY 302	-3.324				
		ALA 367	-3.096				
		ARG 365	-2.631				
		LEU 463	-2.254				

Energy values are in kcal/mol. Residues are sorted highest energy contribution value. Only the most relevant residues are reported.
